# Previous History of Migraine Is Associated With Fatigue, but Not Headache, as Long-Term Post-COVID Symptom After Severe Acute Respiratory SARS-CoV-2 Infection: A Case-Control Study

**DOI:** 10.3389/fnhum.2021.678472

**Published:** 2021-06-28

**Authors:** César Fernández-de-las-Peñas, Víctor Gómez-Mayordomo, David García-Azorín, Domingo Palacios-Ceña, Lidiane L. Florencio, Angel L. Guerrero, Valentín Hernández-Barrera, María L. Cuadrado

**Affiliations:** ^1^Department of Physical Therapy, Occupational Therapy, Physical Medicine and Rehabilitation, Universidad Rey Juan Carlos, Alcorcón, Spain; ^2^Department of Neurology, Hospital Clínico San Carlos, Madrid, Spain; ^3^Headache Unit, Department of Neurology, Hospital Clínico Universitario de Valladolid, Valladolid, Spain; ^4^Neuroscience Research Unit, Institute for Biomedical Research of Salamanca, Salamanca, Spain; ^5^Department of Medicine, Universidad de Valladolid, Valladolid, Spain; ^6^Department of Public Health, Universidad Rey Juan Carlos, Alcorcón, Spain; ^7^Department of Medicine, School of Medicine, Universidad Complutense de Madrid, Madrid, Spain

**Keywords:** COVID-19, migraine, fatigue, post-COVID, anxiety, depression, sleep

## Abstract

**Objective:**

To investigate the association of pre-existing migraine in patients hospitalised and who recovered from severe acute respiratory syndrome coronavirus 2 (SARS-CoV-2) infection with the presence of post-coronavirus disease (COVID) symptoms.

**Background:**

No study has investigated the role of migraine as a risk factor for development of post-COVID symptoms.

**Methods:**

A case-control study including individuals hospitalised during the first wave of the pandemic (from February 20 to May 31, 2020) was conducted. Patients with confirmed previous diagnosis of migraine were considered cases. Two age- and sex-matched individuals without a history of headache per case were also recruited as controls. Hospitalisation/clinical data were collected from hospital medical records. Patients were scheduled for a telephone interview. A list of post-COVID symptoms was systematically evaluated, but participants were invited to freely report any symptom. The Hospital Anxiety and Depression Scale and the Pittsburgh Sleep Quality Index were used to assess anxiety/depressive symptoms and sleep quality. Multivariable conditional logistic regression models were constructed.

**Results:**

Overall, 57 patients with confirmed diagnosis of migraine and 144 non-migraine controls who had recovered from COVID-19 were assessed at 7.3 months (SD 0.6) after hospital discharge. The number of post-COVID symptoms in the migraine group was significantly greater (OR 1.70, 95% CI 1.29–2.25, *P* < 0.001) than in the non-migraine group. Fatigue was significantly more prevalent (OR 2.89, 95% CI 1.32–6.32, *P* = 0.008) in the migraine group. However, no between-groups difference in the prevalence of headache as a post-COVID symptom was detected.

**Conclusion:**

Patients with a history of migraine who recovered from COVID-19 exhibited more long-term fatigue as post-COVID sequelae than those without migraine. Some of the pathophysiological changes associated with migraine could predispose to the occurrence of post-COVID symptoms.

## Introduction

It is now well known that severe acute respiratory syndrome coronavirus 2 (SARS-CoV-2) infection can affect multiple systems, and not only the respiratory system. Indeed, neurological manifestations of coronavirus disease 2019 (COVID-19) are frequent and varied including headaches, dizziness, anosmia, or ageusia (Ellul et al., 2020). A meta-analysis of 14,275 patients reported a headache prevalence of 10.1% (95% CI 8.76–11.49) in patients with COVID-19 (Islam et al., 2020). Headache can occur as an early symptom of COVID-19 at the acute phase (Bolay et al., 2020a) and can even emerge as an isolated symptom (Toptan et al., 2020). Different studies have described that headaches experienced by patients with COVID-19 may resemble migraine or tension-type headache, but no specific phenotype has been observed (López et al., 2020; Porta-Etessam et al., 2020).

Preliminary evidence supports that the presence of headache at the acute phase of SARS-CoV-2 infection is associated with a better course of the disease (Caronna et al., 2020; Gonzalez-Martinez et al., 2021). Furthermore, it has been proposed that the presence of headache at onset of COVID-19 could help clinicians to better characterise the patients; however, data about the relevance of previous headache history in COVID-19 patients is scarce (Belvis, 2020). The SARS-CoV-2 infection disproportionately impacts people with pre-existing medical comorbidities, e.g., diabetes, hypertension, or cardiovascular conditions (de Almeida-Pititto et al., 2020). Migraine is a disabling neurological disease which can be also vulnerable to a negative impact by the COVID-19 outbreak (Szperka et al., 2020). Current data on this topic is heterogeneous, since Al-Hashel and Ismail (2020) reported that 60% of patients suffering from migraine experienced an increase in their frequency of attacks during the worldwide lockdown of March–April 2020, whereas Uygun et al. (2020b) and Parodi et al. (2020) did not find a significant worsening of previous primary headaches due to the pandemic.

Schankin and Straube (2012) observed that patients with pre-existing primary headaches are more prone to develop other secondary headaches. Therefore, it would be expected that migraine sufferers would experience more prolonged and severe headaches related to COVID-19 than those without migraine. In fact, Membrilla et al. (2020) found that patients with pre-existing migraine who suffer from COVID-19 tend to report earlier, longer, and more intense headaches at the onset of the infection than those without pre-existing migraine. It could also happen that migraine itself predisposes patients to suffer more post-COVID symptoms. In fact, the world is suffering a second potential pandemic associated with COVID-19, the “long-haulers” (Rubin, 2020). Current evidence suggests that around 75% of previously hospitalised COVID-19 survivors exhibit at least one post-COVID symptom 2-3 months after the acute phase (Arnold et al., 2020; Carfì et al., 2020; Huang et al., 2021). No previous study investigating the presence of long-term post-COVID symptoms, particularly headache, has considered pre-existing migraine as a risk factor. This study aims to investigate the association of a prior history of migraine and the development of persistent headache and other long-term post-COVID symptoms in individuals hospitalised for COVID-19. We hypothesised that migraine patients infected with SARS-CoV-2 would develop persistent headache as a post-COVID symptom more frequently than those without a history of migraine.

## Materials and Methods

### Participants

A cross-sectional case-control study according to the *Strengthening the Reporting of Observational Studies in Epidemiology* (STROBE) statement was performed (von Elm et al., 2014). From all patients hospitalised due to SARS-CoV-2 infection during the first wave of the pandemic (from February 20 to May 31, 2020) in Hospital Clínico San Carlos, Madrid (Spain), those with confirmed pre-existing migraine history were selected.

Inclusion criteria for the migraine group included: (1) migraine diagnosed by a neurologist according to the International Classification of Headache Disorders, 3rd edition (ICHD-3) criteria (Headache Classification Committee of the International Headache Society (IHS),, 2018); (2) diagnosis of migraine before 50 years of age; (3) at least three attacks in the year prior to infection, and (4) absence of other concomitant primary or secondary headache disorders. The diagnosis of previous migraine was extracted from hospital medical records. In addition, two matched subjects without pre-existing headache history per each case were recruited as controls. Each control was matched by age and sex. If more than two controls per case were available, the selection was done randomly. All participants had been discharged without re-hospitalisation at the time of the study.

All participants had been positively diagnosed of SARS-CoV-2 (ICD-10 code) infection with real-time reverse transcription-polymerase chain reaction (PCR) assay of nasopharyngeal and oral swab samples and/or the presence of consistent clinical and radiological findings at the time of hospitalisation. Patients with medical diagnosis of dementia, delirium, severe psychiatric conditions (or otherwise unable to conduct the interview) were excluded. The study was approved by the Local Ethics Committee of Hospital Clínico San Carlos (HCSC20/495E). Participants were informed of the study and they provided verbal informed consent before collecting any data.

### Procedure

Clinical and hospitalisation data including age, gender, height, weight, pre-existing medical comorbidities, symptoms at hospitalisation, intensive care unit (ICU) admission and days at hospital were collected from hospital medical records.

Participants who agreed to participate were scheduled for a telephonic semi-structured interview by trained healthcare professionals blinded to the patient’s condition (migraineur and non-migraineur). They were also asked to report all the symptoms that they experienced at onset of the infection when they were hospitalised. Additionally, they were asked to report the presence of symptoms appearing after hospitalisation and if the symptom persisted at the time of the interview. It was emphasised that symptoms should have appeared after the infection (post-COVID related symptom). Participants were systematically asked about a predefined list of post-COVID symptoms (i.e., dyspnoea, fatigue, headache, anosmia, ageusia, chest pain, palpitations, diarrhoea, cough, cognitive blunting/brain fog, or loss of concentration), but they were free to report any further symptom that they considered relevant. The clinical description of headache provided by participants was used to describe the phenotype of post-COVID headache as tension-type like or migraine-like headache by two experienced neurologists according to the phenotypic ICHD-3 criteria (Headache Classification Committee of the International Headache Society (IHS),, 2018).

The Hospital Anxiety and Depression Scale (HADS) and the Pittsburgh Sleep Quality Index (PSQI) were used to assess anxiety/depression symptoms and sleep quality, respectively, as both questionnaires can be adequately administered by telephone interview (Hedman et al., 2013). Briefly, the HADS consists of an anxiety symptoms subscale (HADS-A, seven-items) and a depressive symptoms subscale (HADS-D, seven -items). Each item is scored on a Likert scale (0–3) providing a maximum score of 21 points for each scale (Herrmann-Lingen et al., 2011). Although a cut-off score of ≥8 points has shown good sensitivity and specificity (Olssøn et al., 2005), we considered the cut-off scores recommended for the Spanish population (HADS-A ≥ 12 points; HADS-D ≥ 10 points) for determining the presence of anxiety and depressive symptoms, correspondingly (Ministerio De Sanidad Y Consumo, 2008). This questionnaire has been previously used to assess these emotional symptoms of COVID-19 patients during hospitalisation (Deng et al., 2020).

The PSQI was used to evaluate the quality of sleep over the previous month by including 19 self-rated questions assessing the usual bedtime, usual wake time, number of hours slept, and number of minutes to fall asleep (Buysse et al., 1989). Questions are answered on a 4-point Likert-type scale (0–3), and the sum of all answers is transformed into a global score ranging from 0 to 21 points, where higher scores indicate worse sleep quality. A total score ≥8.0 points is indicative of poor sleep quality (Buysse et al., 1989). The PSQI has shown good internal consistency and test-retest reliability (Carpenter and Andrykowski, 1998).

### Statistical Analysis

The statistical analysis was conducted with STATA 16.1 (StataCorp. 2019. Stata Statistical Software: Release 16. College Station, TX: StataCorp LP. United States). Data were presented as mean (standard deviation, SD) or percentages as appropriate. The McNemar and paired Student t-tests were applied to compare proportions and means between migraineurs and non-migraineurs groups. Multivariable conditional logistic regression models were constructed to identify the clinical variables and those corresponding to hospitalisation and post-COVID phases that were independently associated with a prior history of migraine. Adjusted odd ratio (OR) with 95% confidence intervals (95% CI) were calculated. *A priori*, the level of significance was set at 0.05.

## Results

Among 1,000 patients hospitalised due to COVID-19 from February 20 to May 31, 2020, a total of 57 with a confirmed diagnosis of migraine before the infection were identified. Furthermore, 114 age- and sex-matched hospitalised COVID-19 patients without previous migraine history were chosen as controls. Patients with pre-existing migraine had higher weight and BMI than controls (*P* < 0.001) (Table 1).

**TABLE 1:** Demographic and hospitalisation data of coronavirus disease 2019 (COVID-19) patients with and without pre-existing migraine history.

	Migraineurs (*n* = 57)	Non-migraineurs (*n* = 114)
**Age, mean (SD)**	56.5 (17.0)	56.5 (16.0)
**Gender, *n* (%)**		
Male	19 (33%)	38 (33%)
Female	38 (67%)	76 (67%)
Weight, Kg, mean (SD)*	71.5 (12.5)	61.0 (13)
**Height, cm, mean (SD)**	165.0 (10)	165.5 (11)
BMI, kg/cm^2^, mean (SD)*	26.0 (3.5)	22.0 (5.0)
**Smoking status, *n* (%)**		
Active	4 (7%)	12 (10.5%)
None or former	53 (93%)	102 (89.5%)
**Number of medical comorbidities, *n* (%)**		
None	26 (45.6%)	59 (51.7%)
1 or 2	28 (49.1%)	48 (42.1%)
3 or more	3 (5.3%)	7 (6.2%)
**Medical co-morbidities**		
Migraine	57 (100%)	0 (0%)
Hypertension*	16 (28.1%)	15 (13.2%)
Diabetes	6 (10.5%)	10 (8.8%)
Cardiovascular disease	7 (12.3%)	13 (11.4%)
Rheumatological disease	2 (3.5%)	3 (2.6%)
Asthma	4 (7%)	10 (8.8%)
Obesity	4 (7%)	2 (1.7%)
Chronic obstructive pulmonary disease	2 (3.5%)	3 (2.6%)
Other	12 (21%)	23 (20.2%)
**Symptoms at hospital admission, *n* (%)**		
Fever	40 (70.2%)	83 (72.8%)
Dyspnoea	22 (38.6%)	37 (32.5%)
Headache*	22 (38.6%)	19 (16.7%)
Cough	17 (29.8%)	24 (21%)
Myalgia	14 (24.5%)	27 (23.7%)
Anosmia	8 (14.0%)	15 (13.2%)
Diarrhoea	7 (12.3%)	10 (8.8%)
Ageusia	5 (8.8%)	12 (10.1%)
Throat pain	4 (7%)	6 (5.2%)
**Days of hospital stay, mean (SD)**	11.5 (9)	12.5 (13)
**ICU admission**		
Patients admitted to ICU, *n* (%)	4 (7%)	5 (4.4%)
Days in ICU, mean (SD)	2 (1.5)	6 (11)

BMI, body mass index; ICU, intensive care unit; SD, standard deviation.

*Statistically significant differences between cases and controls (*P* < 0.05).

The most common symptoms at hospitalisation due to COVID-19 infection were fever, dyspnoea, headache, and cough. A greater proportion (*P* < 0.01) of migraineurs reported headache as symptom at onset (38.6%) as compared to non-migraineurs (16.5%). No other significant difference in symptoms at the acute phase was observed (Table 1). Eighty-five (49.7%) patients had no comorbidity, 76 (44.4%) had one-two comorbidities, and the remaining 10 (5.9%) reported 3 or more comorbidities. In general, no significant between-groups differences in the number of pre-existing co-morbidities were found. A significant greater proportion (*P* = 0.033) of patients with pre-existing migraine reported comorbid hypertension (28.1%) when compared with those without pre-existing migraine (13.2%). Demographic and hospitalisation data of the population are summarised in Table 1.

Participants were assessed a mean of 7.3 months (SD 0.7) after hospital discharge. At the time of the evaluation, from the total sample only 57 (33.3%) were completely free of any post-COVID symptom, 84 (49.1%) had one or two symptoms, and the remaining 30 (17.6%) had ≥3 post-COVID symptoms. A significantly greater proportion (*X*^2^: 13.636, *P* = 0.004) of patients with pre-existing migraine history reported three or more post-COVID symptoms when compared to those without migraine. In fact, the number of post-COVID symptoms within the migraine group (mean 2.7, SD 1.4) was significantly greater (OR 1.70, 95% CI 1.29–2.25, *P* < 0.001) than the number of post-COVID symptoms in the non-migraine group (mean 1.8, SD 1.45).

**FIGURE 1:**
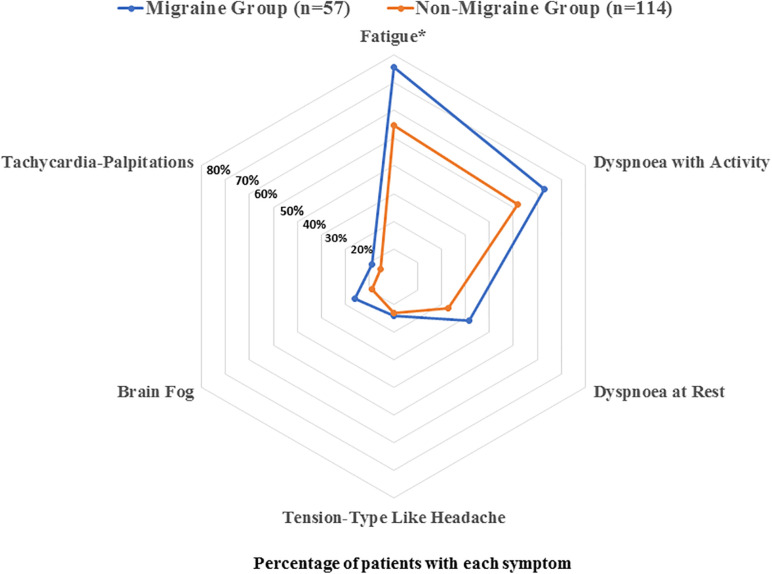
Distribution of the most prevalent post-COVID symptoms (fatigue, dyspnoea with activity, dyspnoea at rest, tension-type like headache, brain fog, and tachycardia) in coronavirus disease 2019 (COVID-19) patients with and without previous migraine history.

Figure 1 graphs the distribution of post-COVID symptoms in patients with and without previous migraine 7 months after hospitalisation. The most prevalent long-term post-COVID symptoms were fatigue, dyspnoea with exercise, dyspnoea at rest, memory loss, and tension-type like headache. A significantly greater proportion (OR 2.89, 95% CI 1.32–6.32, *P* = 0.008) of patients with previous migraine history reported fatigue (75.4%) as compared to those without migraine (54.5%). However, no significant difference in the prevalence of headache as a post-COVID symptom between groups was detected (OR 1.08, 95% CI 0.41–2.87, *P* = 0.869).

There was also no difference in the prevalence of dyspnoea at rest (OR 1.64, 95% CI 0.77–3.50, *P* = 0.193), dyspnoea with activity (OR 1.55, 95% CI 0.80–2.99, *P* = 0.182) between groups (Table 2).

**TABLE 2:** Prevalence of post-COVID symptoms in COVID-19 patients with and without pre-existing migraine history.

	Migraineurs (*n* = 57)	Non-migraineurs (*n* = 114)
Number of post-COVID symptoms, *n* (%)*		
None	10 (17.5%)	47 (41.2%)
1 or 2	32 (56.2%)	52 (45.6%)
3 or more	15 (26.3%)	15 (13.2%)
**Types of post-COVID symptoms, *n* (%)**		
Fatigue*	43 (75.4%)	62 (54.4%)
Dyspnoea with activity	36 (63.2%)	60 (52.6%)
Dyspnoea at rest	18 (31.6%)	26 (22.8%)
Memory loss	10 (17.5%)	22 (19.3%)
Tension-type-like headache	8 (14%)	15 (13.2%)
Cognitive blunting/Brain fog	9 (15.8%)	10 (8.8%)
Tachycardia/Palpitations	5 (8.8%)	6 (5.3%)
Dizziness*	5 (8.8%)	2 (1.8%)
Ocular/Vision Disorders	3 (5.3%)	8 (7%)
Ageusia/Hypogeusia	3 (5.3%)	4 (3.5%)
Anosmia/Hyposmia	2 (3.5%)	4 (3.5%)
Cough	1 (1.8%)	2 (1.8%)
Gastrointestinal disorders-Diarrhoea	1 (1.8%)	2 (1.8%)
Migraine-like headache	0 (0.0%)	3 (2.6%)
**HADS-D, mean (SD)**	5.25 (4.5)	4.5 (5)
Depression (≥10 points), *n* (%)	14 (24.5%)	24 (21.1%)
**HADS-A, mean (SD)**	5.75 (4.5)	4.7 (5.1)
Anxiety (≥12 points), *n* (%)	7 (12.3%)	18 (15.8%)
**PSQI, mean (SD)**	7.2 (4.5)	7.0 (4.5)
Poor sleep quality (≥8 points), *n* (%)	20 (35.1%)	45 (39.5%)

HADS, Hospital Anxiety and Depression Scale (A: Anxiety; D: Depression); PSQI, Pittsburgh Sleep Quality Index; SD, standard deviation.

*Statistically significant differences between cases and controls (*P* < 0.05).

No significant differences in HADS-A (*P* = 0.198), HADS-D (*P* = 0.463), and PSQI (*P* = 0.765) scores were observed between both groups. In fact, no significant association between the presence of pre-exiting migraine with depressive symptoms (OR 1.20, 95% CI 0.58–2.48, *P* = 0.617), anxiety symptoms (OR 0.74, 95% CI 0.29–1.90, *P* = 0.538) and poor sleep quality (OR 0.83, 95% CI 0.43–1.60, *P* = 0.581) was either found (Table 2).

The multivariate analysis revealed that, after adjusting by all variables, both a greater BMI (OR 1.20, 95% CI 1.08–1.34, *P* < 0.001) and a greater number of post-COVID symptoms (OR 1.50, 95% CI 1.09–2.09, *P* = 0.015) were associated with a previous history of migraine.

## Discussion

This study describes, for the first time, the prevalence of long-term post-COVID symptoms in COVID-19 survivors with a confirmed diagnosis of migraine. We observed that patients with migraine who recovered from COVID-19 exhibited more long-term post-COVID symptoms, particularly fatigue, than those without migraine. No differences in anxiety/depressive levels or sleep quality were found between migraineurs and non-migraineurs.

Evidence supports that symptoms at COVID-19 onset are highly heterogeneous, with fever, cough, fatigue, and dyspnoea being the most prevalent (Alimohamadi et al., 2020). We also observed that headache as a symptom during the acute phase of the infection was more prevalent in patients with migraine. Our results agree with those of previous studies, which also found that a history of headache was more common in patients presenting with headache at onset, but, obviously, this was not exclusive (Trigo et al., 2020; Gonzalez-Martinez et al., 2021). In fact, headache of acute COVID-19 is usually perceived qualitatively different from the usual headache experienced by these patients (Uygun et al., 2020). In line with these observations, Planchuelo-Gómez et al. (2020) observed that the presence of pre-existing migraine was not associated with a particular phenotype of headache at onset of SARS-CoV-2 infection; however, they just included 18 patients with pre-existing migraine. Although headache has been previously associated with other neurological symptoms, e.g., anosmia or ageusia (Rocha-Filho and Magalhães, 2020), we did not find such association.

We reported the prevalence of headache as a post-COVID symptom to be 15% in our sample of COVID-19 survivors. Previous prevalence rates on post-COVID headache are slightly heterogeneous. Our prevalence rate was similar to that one previously seen by Carfì et al. (2020), but much higher than rates reported by Arnold et al. (2020) and Huang et al. (2021) and much lower than data reported by Gonzalez-Martinez et al. (2021). Different age, pre-existing comorbidities, previous headache history, time from hospital discharge, or clinical course of COVID-19 disease may explain these discrepancies. Interestingly, previous history of migraine did not influence the development of post-COVID headache, in agreement with Gonzalez-Martinez et al. (2021). Future multicenter studies including large population and conducting a systematically scrutiny of headache as a post-COVID symptom are needed to determine the clinical relevance of this symptom.

Despite not being associated with a higher incidence of persistent headache, a history of previous migraine was associated with a higher number of post-COVID symptoms 7 months after discharge, particularly fatigue, which is really intriguing. The fact that migraine and COVID-19 share common underlying mechanisms would support that this comorbid condition could promote the occurrence of more post-COVID symptoms. The prolonged pro-inflammatory response (cytokine storm) in COVID-19 patients can lead to a rapid hyperactivation of T cells, macrophages, and natural killer cells, and the overproduction of >150 inflammatory mediators (Mulchandani et al., 2021). This cytokine storm can promote an atypical response of the mast cells (Afrin et al., 2020), a dramatic increase of interleukin-6 (IL-6) levels (Coomes and Haghbayan, 2020), and over-expression of the angiotensin-converting enzyme 2 (ACE2) at central and peripheral nervous systems (Sharifkashani et al., 2020). Interestingly, these three mechanisms, i.e., hyper-responses of mast cells (Conti et al., 2019), neuroinflammation by IL-6 (Bougea et al., 2020), and hyper-activation of ACE2 receptors (Gales et al., 2010), also play an important role in migraine.

Since hypertension is comorbid with migraine in almost 30% of individuals (Bigal et al., 2010), some vascular mechanisms associated with SARS-COV-2 pathophysiology, e.g., participation of nucleotide oligomerisation domain-like receptor family pyrin domain containing 3 (NLRP3) inflammasome complex and pericyte dysfunction, may also contribute to the neuro-vasculo-inflammatory mechanisms of migraine (Bolay et al., 2020b). It is possible that these responses lead to hyper-excitability of the trigemino-vascular system throughout different pathways promoting sensitisation mechanisms usually observed in migraine. In such a scenario, SARS-COV-2 infection could activate a cascade of events that, in predisposed patients with pre-exiting migraine, would lead to the development of systemic post-COVID symptoms. Indeed, mechanisms of central sensitisation, a key process for pain generation in migraine, could also play a role in the development of other symptoms, i.e., chronic fatigue in COVID-19 and other post-viral syndromes.

Emotional factors could also be involved. Surprisingly, we did not observe differences in the presence of anxiety/depressive levels and poor sleep quality between migraineurs and non-migraineurs. Nevertheless, the sample size was relatively small, so these results could be associated to a type II error. Perhaps future studies will shed light on the potential underlying mechanisms that explain a higher incidence of post-COVID systemic symptoms in individuals with pre-existing migraine, which could serve to improve the management of these patients.

Seven months after hospitalisation, one of the symptoms that was significantly more frequent among patients with pre-existing migraine was dizziness. However, dizziness and vertigo may be also part of the clinical picture of migraine (Iljazi et al., 2020). Since we collected data cross-sectionally, we cannot determine whether this symptom could be present before infection or not in our sample of migraineurs. Therefore, dizziness could either be a new-onset post-COVID symptom or an exacerbated symptom directly related to migraine.

Although this study includes the largest sample of COVID-19 surviving migraineurs evaluated in the longest follow-up period to date, some limitations should be considered. First, we only included hospitalised COVID-19 survivors, hence, current data could be different in non-hospitalised patients. In addition, it should be recognised that the number of those patients with a clear diagnosis of migraine was relatively small, however, since we aimed to have a higher specificity, only those patients that had been diagnosed by a neurologist were included. Second, we did not collect objective measures, e.g., biomarkers, which can help to characterise the severity of COVID-19 in patients with migraine. Third, data were collected telephonically and not face-to-face. Although headache diagnoses were conducted by trained neurologists according to the ICHD-3 diagnostic criteria (Headache Classification Committee of the International Headache Society (IHS),, 2018), it should be recognised that this procedure has a potential bias well known in population survey studies. In fact, we did not use a headache diary for evaluating the evolution of post-COVID headaches and the analysis was limited to a particular moment of the study. Nevertheless, it should be remarked that studies investigating post-COVID symptoms have used similar methods of recruitment (Arnold et al., 2020; Carfì et al., 2020; Huang et al., 2021). Finally, we collected data cross-sectionally; therefore, we were not able to determine the evolution of headache during the follow-up period after hospital discharge, making it difficult to exclusively attribute to SARS-CoV-2 infection the development of headache 7 months after hospitalisation. New studies phenotyping the clinical features of pre-existing migraine history as well as long-term post-COVID headache and other post-COVID symptoms are needed.

In conclusion, in this study we found that patients with a history of migraine who recovered from COVID-19 exhibited more long-term post-COVID symptoms, particularly fatigue, than those without migraine. However, the presence of headache as a long-term post-COVID symptom was not associated with a prior history of migraine. No differences in anxiety or depressive levels and sleep quality were found between migraineurs and non-migraineurs. Some of the pathophysiological changes associated with migraine could also predispose to the occurrence of long-term symptoms in COVID-19 survivors.

## Data Availability Statement

The raw data supporting the conclusions of this article will be made available by the authors, without undue reservation.

## Ethics Statement

The study was approved by the Local Ethics Committee of Hospital Clínico San Carlos (HCSC20/495E). The patients/participants provided their written informed consent to participate in this study.

## Author Contributions

All authors contributed to the study concept and design. CF-P, VG-M, DG-A, and VH-B conducted literature review and did the statistical analysis. VG-M, DG-A, AG, and MC recruited participants. CF-P, DP-C, LF, and VH-B collected the data. MC supervised the study. All authors contributed to interpretation of data. CF-P, VG-M, and DG-A contributed to drafting the manuscript. All authors revised the text for intellectual content and have read and approved the final version of the manuscript.

## Conflict of Interest

The authors declare that the research was conducted in the absence of any commercial or financial relationships that could be construed as a potential conflict of interest.
